# Atypical Presentation of C. Difficile Infection: Report of a Case with Literature Review

**DOI:** 10.7759/cureus.563

**Published:** 2016-04-08

**Authors:** Salman A Khan, Arooge Towheed, Sibghat Tul Llah, Aref Bin Abdulhak, Nancy R Tilson-Mallett, Alan Salkind

**Affiliations:** 1 Department of Internal Medicine, University of Missouri-Kansas City; 2 Department of Medicine, School of Medicine, University of Missouri-Kansas City

**Keywords:** c.difficile enteritis, lack of diarrhea, leukocytosis

## Abstract

Clostridium difficile (C. difficile) is a gram-positive, obligate, anaerobic spore-forming bacillus first reported by Hall and O'Toole in 1935. It occurs mostly after antibiotic use and invariably presents with watery diarrhea. We describe an atypical presentation of C. difficile in a 64-year-old Caucasian female who presented to the our emergency department with abdominal pain, nausea, and vomiting for one day. A complete blood count revealed leukocytosis 30 x 10^9^/L and a subsequent computed tomography (CT) scan of the abdomen and the pelvis, showed fluid filled small bowel loops consistent with enteritis. Her presentation was unusual for lack of diarrhea, the hallmark of C. difficile infection. She was admitted and treated with oral vancomycin. The polymerase chain reaction (PCR) value in the stool for C. difficile was positive. The patient responded very well: her abdominal pain resolved and leukocyte count normalized after a few doses of vancomycin (125 mg po qid). The patient's progress was followed in our clinic for the last three months.

## Introduction

Clostridium difficile (C. difficile) infection is a common complication after antibiotic therapy [1]. The infection is classically associated with the use of clindamycin, though it is also associated with penicillins, cephalosporins, and flouroquinolones. The incidence of C. difficile infection has increased due to the increased use of antibiotics. Other factors like gastric acid suppression, advanced age, severe illness, enteral feeding, obesity, chemotherapy, gastroenteral surgery, and hematopoietic stem cell transplant are also considered as predisposing factors for C. difficile infection. Furthermore, the increased trend for organ transplant has also contributed to the increased incidence of C. difficile.

The mode of transmission is usually the feco-oral route. Interestingly, C. difficile spores are heat resistant and are not killed by alcohol-containing hand sanitizer. When ingested, the spores survive the acidic environment of the stomach and upon exposure to bile acids, they germinate, multiply, and turn into vegetative cells. The carriers themselves remain asymptomatic but act as a reservoir for contamination of the environment [2]. Antibiotics cause disruption of normal intestinal flora and provide an ideal environment for the growth of the organism [3]. Consequently, the proliferation of C. difficile organisms, once the mucosal barrier is breached, manifests as abdominal pain, watery diarrhea, and leucocytosis. Here, we would like to share an interesting clinical scenario, where a suspected case of C. difficile infection presented without diarrhea with involvement of the small bowel rather than the large bowel. Informed consent was obtained from the patient for this study.

## Case presentation

A 64-year-old Caucasian female presented to the emergency department with diffuse abdominal pain of one day’s duration, for which there were no relieving or aggravating factors, and no relation with meals consumed. It was associated with nausea and one episode of non-bilious non-bloody vomiting. She had a medical history of hypertension, chronic obstructive pulmonary disease, pancreatitis secondary to cholelithiasis that resulted in a pancreatic left colonic fistula, and consequent stricture and bowel obstruction. This complication was managed with transverse loop colostomy, segmental small bowel resection, and adhesion lysis. She was discharged home on a course of sulfamethoxazole/trimethoprim C. difficile,800/160 mg po bid, clindamycin, 300 mg po tid for two weeks, and oral vancomycin, 125 mg po qid for three weeks. After discharge, she did well for six weeks and her average daily colostomy output was 400 ml. She denied any change in frequency or amount of colostomy output, blood, or mucus discharge from the colostomy.

**Figure 1:**
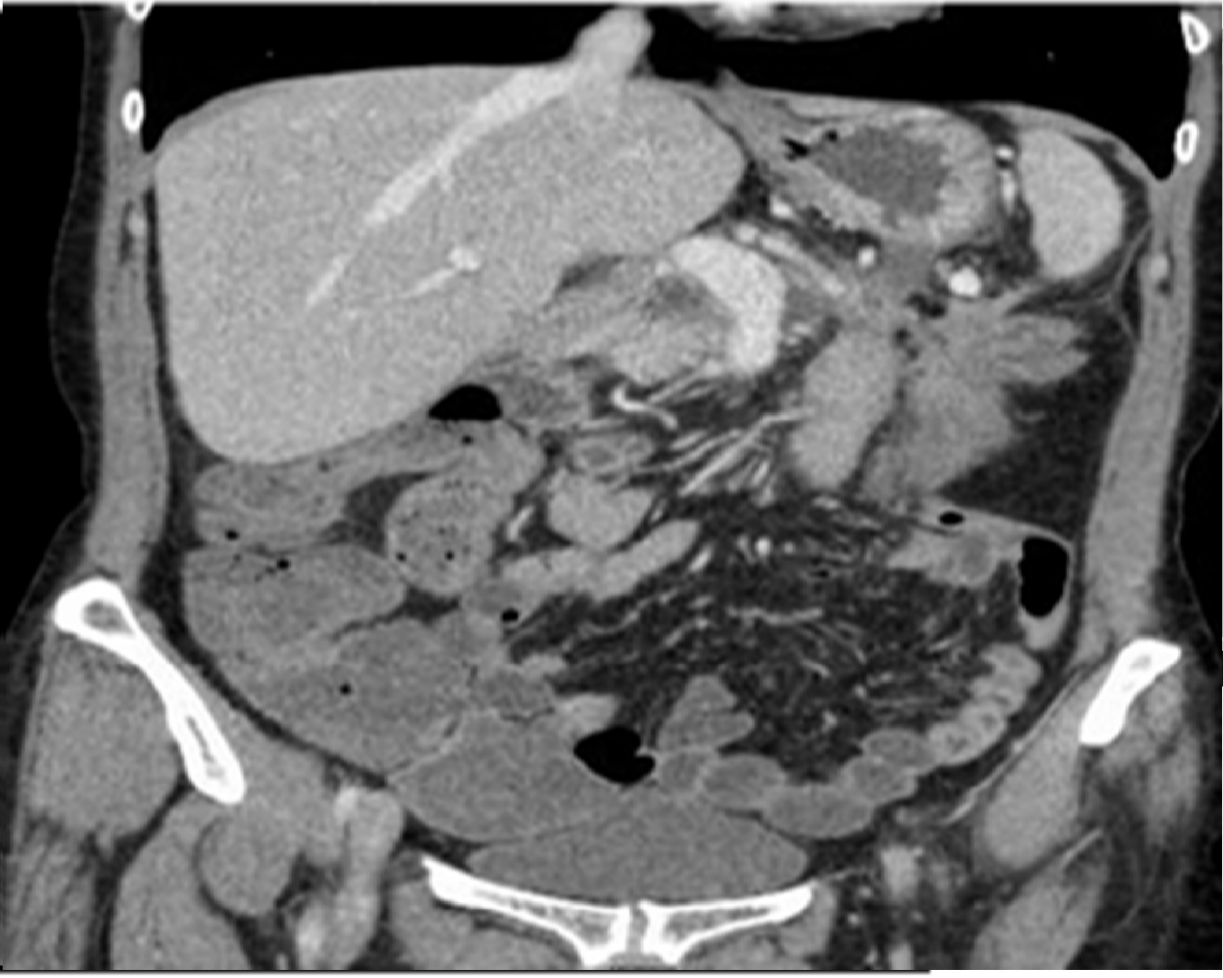
CT Abdomen and Pelvis with IV Contrast Coronal CT images of the abdomen and pelvis demonstrating fluid-filled small bowel loops with mild-moderate wall thickening.

On examination, she was hemodynamically stable and afebrile. Mild tenderness was noticed all over her abdomen; however, no rebound tenderness was elicited. Rest of the systemic examination was unremarkable. The blood work showed a leukocyte count of 30 x 10^9^/L (normal value 11 x 10^9^/L) with predominant neutrophils (94%), hemoglobin 9.4 g/dl (normal value 12-15 g/dl) and platelet 317 x 10^9^/L( normal value 150 x 10^9^/L). Her renal and liver function tests were normal. Blood, urine cultures, and a chest X-ray did not reveal any source of infection; a computed tomography (CT) scan of the abdomen and pelvis showed fluid-filled normal caliber segments of small bowel with minimal ascites, consistent with enteritis. Bowel obstruction was excluded based on CT scan findings (Figure 1). Keeping in view her clinical status she was started on oral vancomycin 125 mg po qid suspecting C. difficile while the result of the work up was awaited. The patient had a remarkable improvement in her symptoms and the white cell count reduced to 13,000 x 10^9^/L the next day. Later on, C. difficile was confimed on stool PCR. She continued to improve with treatment and was discharged home after six days of hospitalization. She is doing well and her progress was followed in our clinic for the past three months.

## Discussion

The index case presented with abdominal pain, nausea, vomiting, and high leukocyte count in the absence of diarrhea, the cardinal feature of C. difficile gastrointestinal infection. Furthermore, the patient had enteritis, another uncommon presentation. Apart from few case reports, most of the cases in literature have not documented high leukocytosis with C. difficileinfection, as was observed in our case. Recent increase in the carrier state of C. difficile infection in tertiary care hospitals, as well as long-term acute care facilities in the United States, has resulted in an increase in the incidence of C. difficile infection. Studies have found that approximately 20% of hospitalized patients are C. difficile carriers and the number increases to around 50% in long-term care facilities. Carriers shed C. difficile in stool, they themselves remain asymptomatic [4], but act as reservoirs to infect others. Clinical presentations have been reported from asymptomatic carrier to fulminant colitis with toxic megacolon [5]. Watery diarrhea is the most common presentation and invariably patients experience 10-15 episodes of watery diarrhea per day associated with lower abdominal pain, fever, and leukocytosis. Fever is usually present in severe C. difficile*-*associated diarrhea. The extracolonic manifestation of C. difficile reported by Jacob, et al. includes enteritis, reactive arthritis, bacteremia, cellulitis/soft tissue infections, empyema, splenic and pancreatic abscess, and prosthetic device infections [6]. Although the pathogenesis of C. difficile enteritis is not well understood [5-6]. It is widely accepted that NAP1/027 is a highly virulent strain of C. difficile. We do not know exactly whether this strain was the culprit in our patient as we did not test for this virulent strain [7].

Regarding C. difficil*e* associated enteritis as observed in our patient, several theories have been proposed. Tsutaoka, et al. describes that after colectomy, the small bowel can acquire a colon-like environment and antibiotic exposure can predispose these patients to develop enteritis [8]. To the best of our knowledge, most cases of C. difficile enteritis reported in the literature were observed in patients with altered bowel anatomy. Kralovich postulated that normal small bowel peristalsis and ileocecal valve function together help to prevent colonization in the small intestine, however after alteration in the bowel anatomy after surgery, the small bowel can be colonized by C. difficile and become susceptible to harboring this infection [9]. Therefore, it is pivotal to have a high clinical suspicion in similar clinical scenarios and order stool PCR for C. difficile. In a given clinical setting a CT scan of abdomen and pelvis may be planned to rule out C. difficile enteritis. Treatment can be individualized as no current guidelines are available for treatment of C. difficile enteritis. Patients in the reported cases have been treated with either oral or IV metronidazole, oral vancomycin or a combination of metronidazole and vancomycin. With increased awareness amongst physicians and better therapeutic strategies, mortality associated with C. difficile enteritis has decreased to 25% compared to 66% reported in earlier series [10]. It has widely been accepted that fecal microbiota transplantation is the gold standard for recurrent, multidrug (vancomycin, metronidazole, fidaxomicin) resistant C. difficile colitis [11].

Nevertheless, with all advancements in the treatment strategies, mortality still remains high in patients with C. difficile infection. High level of suspicion for C. difficile infection in susceptible individuals, and early implementation of infection-directed therapy can improve outcome further. Moreover, there is a need for future prospective studies to understand pathophysiology and risk factors associated with C. difficile infection and development of more acceptable treatment strategies than fecal microbiota transplantation.

We found unique features in our case that include lack of diarrhea, an involvement of the small bowel, and a significantly high WBC count.

## Conclusions

Even with all advancements in the treatment strategies, mortality still remains high in patients with C. difficile infection. A high level of suspicion in susceptible individuals and early implementation of infection-directed therapy can improve outcomes. Moreover, there is a need for future prospective studies to understand the pathophysiology and risk factors associated with C. difficile infection and development of more practically acceptable treatment strategies than fecal microbiota transplantation for disease cure.
